# A Holistic Decision-Making Tool for Canine Chronic Kidney Disease: Navigating Palliative Care and Euthanasia

**DOI:** 10.3390/ani16040669

**Published:** 2026-02-20

**Authors:** Diego Antonio Sicuso, Vito Biondi, Pietro Gambadauro, Michela Pugliese, Angelo Peli, Annamaria Passantino

**Affiliations:** 1 Department of Veterinary Sciences, University of Messina, Via Umberto Palatucci, 98168 Messina, Italy; diego150899@gmail.com (D.A.S.); v.biondi@hotmail.it (V.B.); pietro.gambadauro@studenti.unime.it (P.G.); annamaria.passantino@unime.it (A.P.); 2 Department for Life Quality Studies, University of Bologna, 47291 Rimini, Italy; angelo.peli@unibo.it

**Keywords:** Chronic Kidney Disease (CKD), euthanasia, canine, ethics, animal welfare, Quality of Life (QoL), palliative care, One Welfare

## Abstract

Background: Managing end-of-life decisions in canine chronic kidney disease (CKD) remains one of the most significant challenges in veterinary nephrology, requiring a delicate balance between clinical data and ethical considerations. Objective: This study introduces a novel decision-making algorithm designed to guide clinicians and owners through the complexities of end-of-life care, focusing on the assessment of Quality of Life (QoL) and disease progression. Methods: The tool integrates IRIS staging with a multi-parameter QoL checklist, categorizing patients into three distinct clinical pathways: Tier A (Active Palliative Care), Tier B (Intensive Monitoring/Ambiguous Zone), and Tier C (Compassionate Euthanasia). Results and Discussion: The algorithm defines Tier A as a proactive palliative pathway grounded in systematic clinical evaluation and continuous QoL assessment, aimed at preserving patient comfort and dignity. Across all tiers, QoL functions as a dynamic and central parameter, guiding transitions between clinical pathways in conjunction with objective indicators of disease progression. Tier C delineates the threshold of therapeutic futility, where declining QoL and biological resilience converge, and euthanasia is framed as an ethically appropriate option to prevent refractory suffering. Conclusions: By providing methodological transparency and clear clinical thresholds, this tool facilitates communication between veterinarians and owners. It ensures that palliation is approached with high-standard medical rigor while safeguarding the animal’s dignity when biological resilience is exhausted.

## Simple Summary

Ethical decision-making in veterinary medicine often suffers from subjective bias, particularly in the management of terminal conditions like chronic kidney disease (CKD). This paper presents a structured algorithm that translates clinical scores into actionable ethical tiers. We redefine palliative care as an active medical commitment—focusing on the mitigation of uremic gastrointestinal distress and the prevention of metabolic crises—rather than a passive withdrawal of care. This framework provides a transparent roadmap for clinicians, ensuring that every intervention, from proactive palliation to compassionate euthanasia, is grounded in the principle of non-maleficence and the preservation of the animal’s dignity.

## 1. Introduction

Chronic Kidney Disease (CKD) is one of the most common pathologies affecting geriatric dogs, characterized by a slow and irreversible loss of renal function [1]. Recent insights into the etiopathogenesis of these conditions highlight how initial insults to the nephron, whether caused by toxins, infections, or immune-mediated diseases, lead to a self-perpetuating cycle of inflammation, fibrosis, and functional decline [2]. After an initial “trigger” phase, the kidneys enter a compensatory stage where the remaining functional nephrons increase their activity (hyperfiltration). While this maintains clinical stability temporarily, it eventually leads to a “vicious circle” characterized by glomerular hypertension, inflammation, and progressive hypoperfusion.

**Figure 1:**
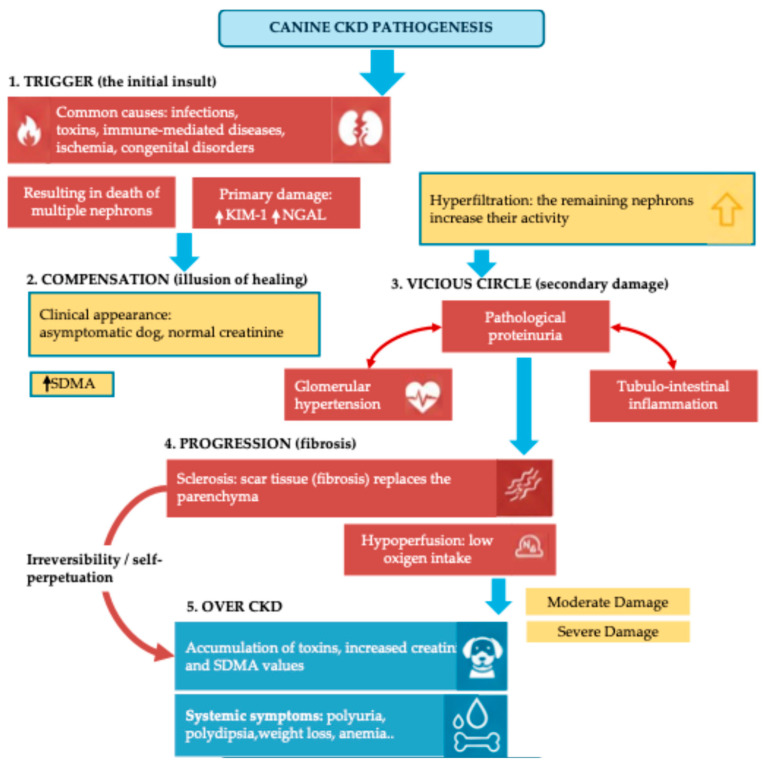
Pathogenesis and progression of Canine Chronic Kidney Disease. The diagram summarizes the five key stages of CKD: (1) Trigger (initial insult); (2) Compensation (hyperfiltration and asymptomatic phase); (3) Vicious Circle (secondary damage including proteinuria and hypertension); (4) Progression (fibrosis and sclerosis of the parenchyma); and (5) Over CKD (clinical appearance of systemic symptoms such as polyuria, polydipsia, and anemia). Diagnostic markers such as KIM-1/NGAL, SDMA, and Creatinine are indicative of the stage of damage.

This pathological progression, which moves from the initial insult to the replacement of functional parenchyma with scar tissue (fibrosis), eventually culminates in overt CKD and systemic uremic symptoms. The various stages of this self-perpetuating cycle and the corresponding clinical markers are illustrated in Figure 1.

The decline in renal function leads to the accumulation of various compounds in the blood, such as urea, creatinine, phosphorus, uric acid, guanidine, and related derivatives. Increased blood concentrations of these uremic solutes promote the uremic syndrome, characterized by multi-organ disorders and cellular dysfunctions [3,4], significantly impacting the patient’s QoL and eventually necessitating the consideration of euthanasia. Additionally, CKD patients have been reported to have reduced gut motility, increased intestinal permeability, bacterial overgrowth, bacterial translocation, and intestinal inflammation [5].

CKD arises from a variety of etiologies and underlying pathologies, such as diabetes, hypertension, glomerular diseases, and genetic factors. These factors contribute to the variability of the disease in each patient, and the presentation may also differ depending on the species affected [6,7]. As a result of that individual variability, making diagnoses and treatment can be challenging. Although initially it was suggested that acute kidney injury (AKI) and CKD should be diagnosed differently in dogs and cats [6], it has recently been proposed that one can lead to the other, as observed in human cases [8,9].

The guidelines of the International Renal Interest Society (IRIS) provide a standardized framework for diagnosing CKD in dogs and cats, categorizing the disease into four stages based on clinical signs and physical examination findings [10].

Stage 1 is characterized by confirmed kidney disease despite circulating uremic toxins remaining within normal reference ranges. In dogs, serum creatinine concentrations are typically below 1.4 mg/dL, whereas symmetric dimethylarginine (SDMA) is persistently increased above 14 µg/dL. Clinical signs directly attributable to renal dysfunction are generally absent; however, other findings—such as persistent proteinuria, abnormal renal imaging results, or a history of acute kidney injury—may be evidence of underlying renal disease.

Stage 2 is characterized by a mild accumulation of nitrogenous waste products in the bloodstream. Serum creatinine concentrations range from 1.4 to 2.0 mg/dL, and SDMA levels fall between 18 and 35 µg/dL. In this case, the kidneys’ excretory capacity is considerably compromised and, despite clinical signs that might still be subtle, polyuria and polydipsia can occasionally be observed. Management often focuses on renal protective strategies to mitigate disease progression.

Stage 3 is characterized by a marked reduction in glomerular filtration rate, resulting in moderate to severe azotemia. Serum creatinine concentrations typically range from 2.1 to 5.0 mg/dL, while SDMA values fall between 36 and 54 µg/dL. Clinical signs become more evident and diverse, commonly including anorexia, weight loss, lethargy, vomiting, and halitosis, all indicative of progressive systemic uremia. At this stage, therapeutic goals focus on mitigating clinical manifestations and preserving the remaining renal function.

Stage 4, the terminal phase of chronic kidney disease, is defined by profound renal dysfunction and severe azotemia. Serum creatinine levels exceed 5.0 mg/dL, with SDMA concentrations typically greater than 54 µg/dL. Affected dogs exhibit severe and often debilitating clinical signs, such as persistent vomiting, marked cachexia, dehydration, uremic stomatitis, and gastrointestinal ulceration. In this advanced stage, treatment strategies are primarily based on intensive supportive care and palliative management to optimize the patient’s QoL.

In this context, it is crucial to define palliative care not as the omission of treatment, but as a proactive and comprehensive medical approach [11]. Unlike therapeutic abandonment, palliative care in CKD involves active interventions, such as tailored hydration, nutritional support, and pain management, aimed at optimizing the patient’s comfort and dignity when a cure is no longer the clinical goal.

Given this progressive, irreversible, and degenerative nature of CKD, especially in its advanced stages (IRIS Stage 3 and 4), which leads to a gradual decline in organ function, veterinarians and pet owners inevitably face difficult decisions regarding the continuation or cessation of treatment. When therapeutic interventions no longer improve or maintain QoL and clinical signs cause significant suffering, euthanasia arises as a compassionate, albeit difficult, option [12].

While the IRIS provides a universally recognized framework for clinical staging and pharmacological management, a significant gap persists in current veterinary practice. There is a lack of structured, ethical tools that bridge the divide between physiological data and the holistic assessment of animal welfare during these critical end-of-life transitions. Standard clinical guidelines [10] are predominantly focused on biochemical parameters, often leaving clinicians and owners to navigate the emotionally taxing shift from palliative care to euthanasia without a standardized, evidence-based roadmap. This absence of a formal decision-support framework frequently results in “decisional conflict”, potential caregiver burnout, and the ethical risk of prolonging suffering beyond the threshold of therapeutic futility.

The ethical debate surrounding euthanasia in these cases revolves around several key themes that are necessary to inform correct decision-making. First, the assessment of QoL is paramount; clinicians must determine if suffering justifies euthanasia or if palliative interventions can still mitigate distress to maintain a balance between prolonging life and avoiding unnecessary pain [13].

Second, owner responsibilities pose a significant challenge as the desire to maintain the human–animal bond must be balanced with the duty to prevent further suffering [14].

Third, the choice between palliative care and euthanasia requires constant reappraisal; although fluids and diet can prolong life with dignity for months, they may become insufficient if the dog’s condition worsens rapidly [15].

Fourth, broader animal welfare principles suggest that prolonging a state of constant suffering is contrary to respect for life, and premature euthanasia without adequate trials of treatment must also be avoided [16,17].

Finally, veterinary guidance remains the cornerstone of this process, providing the professional perspective needed to translate medical prognosis into compassionate action [18,19].

Based on these considerations, the present study aims to provide a structured decision-making framework through a tiered algorithm (Tiers A, B, and C) to guide veterinarians through the critical transition from active treatment to palliative care, and ultimately, to compassionate euthanasia in dogs with CKD. This tool aims to standardize end-of-life care by ensuring that every clinical decision is grounded in the principles of *One Welfare* and guided by the patient’s best interests.

### Legislative Framework of Euthanasia in Dogs

Euthanasia in companion animals lacks a unified international regulatory framework. The World Organization for Animal Health (WOAH) provides general guidance through its Terrestrial Animal Health Code [20], emphasizing that euthanasia should be performed humanely to minimize pain, distress, and suffering. Based on these international recommendations, each country develops its own national legislation, often in coordination with veterinary professional bodies. In Italy, the decision to perform euthanasia is not explicitly regulated by a specific law for companion animals but falls under a broader legal and ethical framework. The only partial legal reference is Law no. 281/1991 [21], which primarily addresses stray populations, permitting euthanasia only for stray animals that are ‘seriously or incurably ill’ or proven to be dangerous. It does not, however, establish broader legal criteria applicable to owned animals. In this legislative vacuum, the Italian Veterinary Code of Ethics [22] offers general guidance, stating that euthanasia should be considered only when it represents the sole option to prevent unnecessary suffering, while always respecting the animal’s dignity.

This normative gap often places veterinarians in a legal and ethical “gray area”, particularly in complex or borderline clinical cases. Without detailed protocols, the burden of decision falls entirely on the veterinarian’s professional judgment and ethical sensitivity. This exposure can lead to external pressure from pet owners or contribute to therapeutic obstinacy, the continuation of treatment despite a poor prognosis and declining QoL.

Given that advanced CKD frequently leads to significant clinical deterioration and compromised QoL, it is essential to establish objective criteria to guide these difficult clinical decisions. To address this need, we propose a conceptual, structured decision-support checklist. Designed to assist both veterinarians and pet owners in systematically evaluating the patient’s condition, this tool facilitates the identification of indicators suggestive of poor welfare. The ultimate objective is to minimize unnecessary therapeutic persistence and to provide ethically grounded recommendations, positioning euthanasia as a compassionate and appropriate intervention when palliative care is no longer sufficient.

## 2. Materials and Methods

### Development of the Checklist for Assessing QoL in Dogs with Chronic Kidney Disease (CKD)

To provide an objective basis for the tiered algorithm, a specialized checklist was developed integrating traditional clinical markers with psychometric indicators of animal welfare, based on models such as the “Five Domains” and the Villalobos HHHHHMM scale. The checklist is designed to be filled out conjointly by the veterinarian and the owner, ensuring that both clinical reality and the dog’s daily experience at home are represented. It was developed through a two-step methodological approach (phase 1 and phase 2) to ensure clinical accuracy and ethical grounding.

Phase 1: Domain Identification and Structure

The initial framework was developed through a narrative review of the scientific literature on canine chronic kidney disease (CKD), palliative care, geriatrics, shared decision-making models, and existing animal welfare assessment tools, to identify key domains and relevant indicators for evaluating QoL in patients affected by chronic or terminal conditions.

From this phase, six preliminary domains emerged: general clinical condition, pain and discomfort, food and water intake, social and environmental interaction, response to treatments, and signs of physical and psychological distress.

Based on these domains and the HHHHHMM scale (Hurt, Hunger, Hydration, Hygiene, Happiness, Mobility, more good days than bad) by Villalobos [23], we created a specific checklist (Table 1) to analyse and evaluate QoL in dogs with CKD. Specifically, we had considered two types of indicators:

(i) Indirect welfare indicators: 11 indicators, divided into (8) general clinical indicators, (1) specific CKD clinical indicator based on IRIS staging, (2) Owner’s responsibility indicators.
(ii) Direct welfare indicators: 2 indicators (Happiness/Environmental interest and Positive vs. Negative days).

Phase 2: Content Validation

To ensure the clinical specificity of the tool for CKD, the proposed criteria were validated through consultation with a multidisciplinary panel of five experts. This process followed an informal, structured consensus approach. Panel members were selected based on their recognized expertise in the following fields:

- Nephrology: to ensure alignment of the indicators with IRIS staging and disease progression.
- Internal Medicine: to assess the influence of comorbidities and multi-organ dysfunction.
- Anesthesiology: to support the accurate evaluation and management of pain and discomfort.
- Animal Welfare: to provide a robust bioethical framework for end-of-life decision-making.

They were engaged in iterative reviews of the indicators.

Simultaneously, particular attention was paid to the clarity and accessibility of the language, ensuring the tool was easy to understand for pet owners and effectively supported a genuinely shared decision-making process. The checklist was designed for use during clinical consultations, home-based follow-ups, and at key milestones in disease progression.

Each indicator was scored from 0 to 2 points, where

- 0 points indicate a severe problem or very poor condition related to that specific aspect (e.g., severe pain, no appetite, or extreme distress);
- 1 point indicates a moderate issue or some impairment, meaning the dog is affected, but the condition is manageable or only partially impacts QoL;
- 2 points indicate a good or no loss condition for that indicator, showing minimal or no negative impact on the dog’s QoL.

The scoring system (0–2 points per indicator) was categorized into three groups based on the patient’s QoL and the efficacy of palliative interventions:

(i) 20–26 Points (Good QoL): Tier A—Minimal negative impact; recommended action is to continue palliative care and regular monitoring.
(ii) 14–19 Points (Reduced but acceptable QoL): Tier B—Ambiguous condition; requires frequent reassessment and intensive communication between the veterinarian and the owner.
(iii) 0–13 Points (Severe impairment of QoL): Tier C—High levels of suffering or treatment futility; euthanasia should be considered an ethical and compassionate priority.

It is important to note that these scoring thresholds are primarily conceptual and based on the collective expert judgment of the authors. These cut-offs are intended as provisional clinical benchmarks to facilitate shared decision-making rather than empirically validated absolute values.

## 3. Results

The following indicators are based on clinical observations, behavioral assessments, and widely accepted ethical principles in veterinary care. They are not merely data points; each one reflects a meaningful aspect of the animal’s life and care context, considering the physical, psychological, and emotional needs of both the dog and its owner. The goal is to provide a clear and compassionate framework to support difficult decisions, including the consideration of euthanasia when necessary.

1. Pain and Discomfort

This category evaluates whether the dog is in pain and if the pain can be effectively managed. A dog showing vocalizations, a rigid posture, or signs of anxiety may be suffering. If the pain is continuous and unmanageable, it violates the ethical principle of non-maleficence, to not harm, and may ethically justify euthanasia. Clinically, CKD can cause painful conditions such as oral ulcers or muscle cramps.

2. Hunger and Nutrition

This assesses whether the dog eats voluntarily and maintains a healthy weight. Prolonged anorexia and muscle wasting (cachexia) are signs of systemic suffering. Forcing a dog to eat can be considered therapeutic overkill and ethically questionable. Clinically, anorexia is common in advanced CKD due to uremic nausea, gastritis, or metabolic disturbances.

3. Hydration

This evaluates the dog’s ability to stay hydrated without constant medical intervention. Dogs who need ongoing fluid therapy are in a severe stage of disease, possibly incompatible with a dignified life. Chronic dehydration leads to systemic discomfort and is typical in later CKD stages.

4. Hygiene and Incontinence

This category assesses whether the dog can keep itself clean or frequently soils itself due to urinary or fecal incontinence. The loss of personal hygiene impacts both physical comfort and dignity. Clinically, incontinence may result from polyuria, uremic neuropathy, or muscle weakness.

5. Happiness/Environmental Interest

This explores the dog’s emotional and behavioral engagement: does it show pleasure, curiosity, interaction with people, or the environment? Persistent apathy suggests psychological suffering or depression. When a dog “is no longer itself,” compassionate euthanasia may be justified. Medically, this can be linked to uremic encephalopathy or chronic discomfort.

6. Mobility

This assesses whether the dog can stand, walk, and move without severe pain. Mobility is essential to QoL, representing autonomy and freedom. Loss of mobility can lead to frustration and dependency. In CKD, reduced mobility can result from anemia, muscle weakness, or electrolyte imbalances.

7. Comorbidity and Multimorbidity Management

Patients with CKD often present with a variety of clinical signs that may overlap with or be exacerbated by concomitant pathologies (e.g., degenerative joint disease, cardiovascular disorders, or endocrine imbalances). It is essential to recognize that not all comorbidities impact the patient’s prognosis equally. The clinical evaluation must focus on the severity of the symptoms, the specific organ systems involved, and, crucially, the potential for therapeutic conflict. A comorbidity is considered high impact if its treatment requires medications that are nephrotoxic or if the management of the CKD (such as aggressive fluid therapy) poses a risk to another compromised system (such as the heart). Therefore, the assessment should not merely count the number of diseases but weigh how these concurrent conditions limit the available therapeutic window and increase the overall clinical burden on the patient.

8. Age-Related Prognostic Indicators

Age is a pivotal factor in the holistic assessment of the canine patient, particularly when balanced against the progression of renal failure. According to a recent study [24] analyzing factors related to canine longevity and mortality in Italy, three distinct age ranges have been identified that correlate with significantly higher probabilities of mortality. In clinically ambiguous scenarios, where the IRIS stage or physical symptoms might not provide a definitive path forward, age evaluation serves as a critical prognostic indicator. It is not simply a chronological measure but a proxy for “biological reserve”. Clearly, this indicator must be interpreted by accounting for confounding factors such as breed and body size, as life expectancy varies significantly in relation to these variables [24]. Older patients in the terminal age brackets often exhibit increased frailty and a diminished capacity to recover from uremic crises. Consequently, age becomes a decisive factor in the ethical decision-making process regarding euthanasia, helping to distinguish between a manageable chronic condition and the natural end-of-life phase where palliative efforts may no longer sustain an acceptable QoL.

9. Positive vs. Negative days

This subjective category evaluates the balance between good and bad days over the past week. A positive day is defined by the maintenance of ethological behaviors, social responsiveness, and spontaneous nutritional intake. Conversely, a predominance of negative days, characterized by persistent lethargy, anorexia, or social withdrawal, indicates a significant and sustained compromise in the animal’s welfare. It shifts the clinical focus from a single cross-sectional snapshot to a temporal balance of life experiences. When the frequency of negative days consistently outweighs the positive, it serves as a critical indicator that the patient has reached a welfare threshold, providing a robust justification for the transition from palliative care to euthanasia.

10. Response to Treatment

This category observes whether palliative treatments (medications, fluids, diet) still improve the dog’s condition. If treatments no longer help, it may indicate the disease has entered a terminal stage. Continuing care in this context may be futile or ethically unjustifiable.

11. Clinical Prognosis (IRIS Stage)

The IRIS staging system classifies CKD severity. However, for QoL assessment, staging must be correlated with clinical symptoms. While IRIS Stages I-III often allow long-term management, Stage IV represents a terminal phase characterized by severe azotemia and often refractory clinical signs. The presence of terminal complications, such as uremic encephalopathy (manifesting as neurological deficits or altered mentation) or systemic hypertensive damage, indicates a transition from chronic management to an end-of-life state. In this context, IRIS staging serves as a critical objective biomarker: a Stage IV diagnosis combined with refractory uremic symptoms provides a strong scientific basis for discussing euthanasia, as the probability of restoring homeostatic balance is statistically negligible.

12. Home-Care Feasibility and Suitability Index

This index evaluates the intersection between a patient’s clinical requirements and the practical sustainability. It supports clinical decision-making by distinguishing cases suitable for home-based management from those requiring inpatient hospitalization. While it is acknowledged that this indicator cannot encompass the full spectrum of variables influencing clinical admission—such as socioeconomic factors [25], the owner’s technical manual skills [26], or the psychological impact of separation [27]—it serves as a critical framework for shared decision-making. It ensures that the treatment plan remains ethically and logistically aligned with the best interests of both the patient and the caregiver, identifying the threshold where home management is no longer viable.

13. Emotional wellness of the owner

This category considers the emotional state of the owner. If they are emotionally exhausted (*burned out*), they may be unable to provide proper care, which directly affects the dog’s QoL. Ethically, the owner’s inability to cope is not merely a human concern but a welfare risk for the dog; inconsistent care can lead to uncontrolled pain, dehydration, and suffering. A scientific assessment of QoL must, therefore, acknowledge that a sustainable treatment plan requires a functional human–animal bond. When the caregiver’s emotional or physical resources are depleted, the resulting “ethical fatigue” may compromise the patient’s dignity and the quality of palliative support provided.

## 4. Discussion

The assessment tool developed in this study demonstrates that QoL in dogs with CKD cannot be adequately captured by clinical markers alone. Although laboratory parameters such as creatinine, blood urea nitrogen (BUN), and SDMA remain crucial for staging the disease, they offer limited insight into the animal’s lived experience. By incorporating indicators such as emotional responsiveness, mobility, hygiene, and pet owner well-being, the tool provides a more holistic and ethically grounded framework for QoL assessment. This approach aligns with the One Welfare concept [28], emphasizing the interconnected well-being of animals, owners, and veterinarians.

Deciding whether to perform euthanasia, especially when the patient is suffering from a chronic degenerative disease, is a very complex issue in veterinary medicine. In addition to purely clinical aspects such as the assessment of QoL, animal welfare, and the implications of prolonged palliative care, it is also crucial to consider collateral factors like owner responsibility and veterinary guidance. The checklist proposed in this study enables the evaluation of QoL in dogs with CKD, while also addressing key themes such as animal welfare, the balance between palliative care and euthanasia, owner responsibility, and veterinary guidance.

Animal welfare is the topic closely associated with the assessments of the first seven indicators on the checklist, which are designed to evaluate the three core physical states making up animal welfare (physical, mental, and activity status) This multidimensional evaluation aligns with the Five Domains model of animal welfare proposed by Mellor and Beausoleil, which emphasizes the importance of both minimizing negative experiences and promoting positive welfare states across physical and affective domains [29]. Specifically, indicators 1 and 5 measure animal welfare, focusing on the mental state of the patient by analyzing their ability to perceive and tolerate pain (pain and discomfort), and their curiosity or interaction with people and/or the environment. Indicators 2, 3, and 4 assess the physical state by examining the patient’s perception of hunger, ability to feed themselves, stay hydrated, and clean themselves. Indicators 6 and 9 address the activity domain by evaluating the dog’s ability to stand, walk, or move without experiencing severe pain. They also provide an overall evaluation of how active and engaged the dog is, and how many days per week they are eating. Finally, indicator 7 evaluates the presence or absence of other diseases over CDK, and whether those influence patient health.

“Palliative care vs. euthanasia” focuses on general clinical signs in patients that reflect the effectiveness of palliative care. The entire checklist aims to assess the patient’s QoL, which is included in the total score in relation to the effectiveness of palliative care. Based on the score, the patient is placed in one of three different groups associated with either high treatment effectiveness (score from 20 to 26), no response (score from 0 to 13), or an ambiguous condition requiring discussion between the veterinarian and the owner (score from 14 to 19). Furthermore, a direct indicator on the checklist has been included (indicator 10) to perform a specific assessment of the effectiveness of palliative care on the patient. In this way, “Palliative care vs. euthanasia” represents the most important topic on which the checklist is based. Focusing on middle score cases (14–19), an important aspect to emphasize is the owner’s willingness/ability to continuously support the necessary care for their animal, which involves commitment not only from an emotional standpoint but also in terms of the actual availability of resources—the latter understood as time and money. This willingness is a multidimensional variable encompassing emotional resilience, financial stability, and logistical feasibility. The efficacy of long-term palliative care for CKD is contingent upon the owner’s ability to provide consistent home-based medical support (e.g., fluid administration, specialized nutrition) and their proximity to emergency veterinary facilities. Because these socio-economic and psychological factors are inherently subjective, they must be integrated into the prognostic assessment, as an unsustainable care plan directly compromises the patient’s welfare. Furthermore, a critical clinical caveat must be applied to the dynamic interpretation of scores. A patient who initially presents in the high-score range (20–26 points) but demonstrates a rapid decline to the intermediate range (14–19 points) during follow-up, despite aggressive supportive therapy, represents a high-risk scenario. In such cases, the prognostic trajectory (the rate of decline) is often more significant than the absolute score. In these instances, euthanasia might be anticipated to avoid the risk that, given the rapid and progressive decline manifested between one assessment and the next, the animal could worsen too quickly to allow for its complete welfare to be protected in time.

Owner responsibility is another key issue in QoL assessment for dogs with advanced CKD (IRIS stages III and IV). The owner must be able to accept the unfavorable prognosis, separate their desire not to “let go” of the animal from the clinical reality, and make decisions that avoid prolonged suffering, even if this means choosing euthanasia. This phase often involves a considerable emotional burden and can cause moral disorientation, especially when the dog experiences intermittent periods of apparent lucidity that alternate with critical phases. Therefore, the checklist also aims to take this fundamental aspect into account when deciding whether to resort to euthanasia. By assessing the animal’s welfare indicators (indicator 1 to 9), those associated with the IRIS clinical staging (11), and those related to the effectiveness of palliative care (10), the aim is to provide owners with concrete data on the condition of their pets, thus making them more aware. Finally, indicator 13 assesses the emotional state of the owner in relation to their pet’s clinical condition.

“Veterinary Guidance” is the final key component of the checklist, although it is not tied to a specific indicator. Instead, it is represented by the compilation process of the checklist itself. Veterinary guidance is an essential part of the treatment and ethical decision-making process for dogs with CKD. Thanks to their clinical and scientific expertise, veterinarians are competent and qualified to accurately assess the stage of the disease (for example, by following the IRIS staging system), monitor clinical progress, and interpret signs of suffering or improvement. Through empathetic communication, veterinarians support owners in interpreting their pet’s QoL, helping them to distinguish between a period of effective disease management and a time when treatment becomes ineffective and risks becoming futile. In addition, the veterinarian acts as a mediator between the owner’s emotional aspect and the animal’s welfare, guiding them through difficult decisions such as euthanasia and ensuring that they are made in an informed and respectful manner. In aligning clinical practice with ethical standards, the British Veterinary Association (BVA) emphasizes the importance of mitigating anthropomorphic projections regarding life expectancy. A critical distinction in veterinary bioethics is that animals are generally understood to lack future-oriented cognition; they experience life “in the present” without the teleological hopes, ambitions, or future-directed desires characteristic of human psychology [30]. Consequently, the clinical focus must remain strictly on the animal’s current quality of experience rather than a quantitative extension of life that lacks qualitative value. Based on this, the BVA proposes a shift from a binary model of “justified versus unjustified” euthanasia toward the concept of “contextually justified euthanasia”. This framework recognizes that clinical decisions do not occur in a vacuum but within a complex ecosystem of stakeholder interests. It necessitates a utilitarian harm-benefit analysis that weighs the interests of the patient, the owner, and the broader social context. When a caregiver is unable to pursue advanced palliative options due to legitimate constraints—such as financial limitations, logistical hurdles, or the absence of local specialized facilities—the patient’s “damage-to-benefit” ratio is significantly altered. In resource-limited environments where alternative interventions (e.g., rehoming or institutionalized care) are unfeasible, the failure to intervene leads to an escalation of cumulative suffering. In such instances, euthanasia may be ethically prioritized as the most humane outcome, as the benefit of alleviating certain distress outweighs the maintenance of a life characterized by unmitigated decline.

The veterinary professional serves as the primary guardian of these legal and ethical benchmarks, ensuring that all end-of-life procedures are performed according to the criteria of humanity and respect. Furthermore, the veterinarian’s role as an educator is vital; by providing evidence-based guidance on home-care strategies and proactive palliative measures, clinicians empower owners to navigate the trajectory of CKD with clinical clarity and ethical confidence.

**Figure 2:**
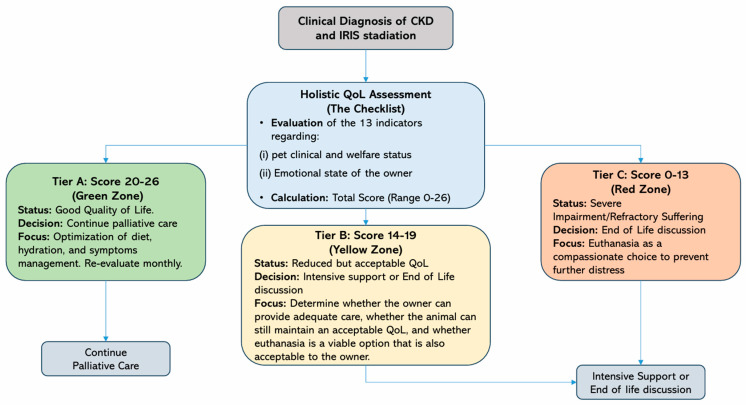
Comprehensive decision-making algorithm for the management of dogs with chronic kidney disease (CKD). The tree integrates clinical IRIS staging with the expert-reviewed QoL checklist scores. The three distinct scoring tiers (0–13, 14–19, 20–26) offer a standardized framework that helps clinicians guide owners in deciding whether to continue palliative care or transition to euthanasia, ensuring an ethical and holistic “One Welfare” approach.

Considering the considerations discussed above, a Decision Tree may serve as a valuable tool to guide veterinarians and owners through the complex decision-making process in dogs with CKD. The proposed decision-making algorithm (Figure 2) outlines a structured clinical–ethical framework that integrates objective scientific evidence with the practical challenges of end-of-life management. Its methodological design is organized into sequential steps that translate clinical assessment into a clear and actionable operational strategy.

Its logic is based on several critical methodological steps.

*Clinical Baseline and Dynamic Trajectory Analysis*: The process originates from IRIS staging, which provides an objective pathophysiological severity parameter, essential for patients in Stages III and IV. However, the model transcends static point-in-time diagnosis by emphasizing the prognostic trajectory (the rate of decline). A rapid transition between score ranges—for instance, a sudden drop from Tier A (high-score range) to Tier B (intermediate range) despite aggressive medical support—is identified as a high-risk welfare indicator. This dynamic approach allows the clinician to anticipate an imminent welfare crisis, favoring proactive euthanasia management over emergency interventions dictated by acute suffering.

*The Three-Tier Scoring Logic*: Integrating the expert-reviewed checklist within the Decision Tree allows for a standardized categorization of the patient’s status:

- Tier A (Score 20–26): Represents a phase of stable welfare, in which the “benefit-to-harm” ratio of palliative measures remains favorable and supports the continuation of care. At this stage, the palliative pathway represents an active shift in clinical focus: from prolonging life to optimizing the quality of the patient’s present experience.

Palliative care in Tier A should not be interpreted as therapeutic withdrawal or passive non-intervention. Rather, it is a proactive and comprehensive medical strategy aimed at preserving comfort, dignity, and a life perceived as worth living when curative treatment is no longer the primary goal.

This tier requires frequent reassessments to ensure that pharmacological and supportive interventions continue to provide a net benefit to the patient’s wellbeing. Clinical management is centered on active symptom control, targeting the main physiological drivers of suffering, including: (i) active control of uremic nausea and vomiting, through targeted pharmacological support (e.g., antiemetics and gastroprotectants) to reduce distress associated with uremic toxins; and (ii) individualized hydration strategies (e.g., subcutaneous or intravenous fluid therapy) to prevent metabolic distress and maintain internal homeostasis.

Through continuous monitoring and therapeutic adjustment, Tier A ensures that palliation remains a high-standard medical commitment, focused on stabilizing the patient’s condition and ensuring that the benefits of treatment consistently outweigh the burdens of disease.

- Tier B (Score 14–19): Defines an “ambiguous zone” of clinical uncertainty, characterized by inconsistent therapeutic response. Methodologically, this tier represents a critical “gray area” where the animal’s biological resilience begins to fade and the response to active palliative treatments becomes inconsistent or partial. In this phase, the decision-making process requires intensified clinical monitoring and frequent ethical reappraisal. The clinician and the owner must determine whether the patient is experiencing a temporary crisis that can be stabilized with intensive support or if they are witnessing an irreversible decline. This stage mandates transparent communication regarding the sustainability of care, acknowledging that if the animal does not stabilize, the transition to Tier C becomes the most ethical progression to avoid a state of prolonged distress.
- Tier C (Score 0–13): Identifies the threshold of therapeutic futility, where severe impairment of QoL and refractory suffering are predominant. At this stage, clinical signs of advanced uremia, such as neurological deficits (uremic encephalopathy), intractable vomiting, or severe cachexia, indicate that the animal’s internal homeostasis can no longer be maintained and its dignity can no longer be preserved through medical means. In alignment with the ethical principle of non-Maleficence, the tool recognizes that continuing treatment in this range would constitute therapeutic obstinacy. Consequently, euthanasia is presented not as a failure of care, but as a compassionate and ethically mandatory medical intervention. The focus shifts entirely to preventing further unnecessary distress, ensuring that the animal’s life concludes with respect and without the escalation of cumulative suffering.

To clarify the operational distinction between these stages and provide the clinician with a practical roadmap. Table 2 summarizes the medical objectives and the expected interventions for each Tier.

“One Welfare” Integration and the Role of the Veterinarian: The model innovatively integrates “One Welfare” principles by formally including an assessment of the owner’s resources and emotional well-being. Recognizing that caregiver burnout or socio-economic limitations can compromise the consistency of care—exposing the dog to direct risks such as uncontrolled pain or dehydration—these human factors become an integral part of the final clinical recommendation.

Ultimately, veterinary guidance remains the pivot of this decisional ecosystem. By synthesizing clinical data, Tier-based scores, and owner feedback, the professional is empowered to recommend the most humane course of action. This holistic approach ensures that the decision-making process is not based on isolated data points but on a comprehensive clinical and ethical trajectory that prioritizes the animal’s QoL while supporting the owner through an emotionally taxing transition.

## 5. Study Limitations and Future Directions

Despite the structured approach used to develop this decision-making tool, some limitations must be acknowledged. First, the scoring thresholds and clinical tiers defined in this study are currently based on conceptual frameworks and expert consensus rather than retrospective or prospective empirical data. While these cut-offs provide a necessary starting point for clinical guidance, their sensitivity and specificity in predicting survival times or welfare outcomes have not yet been statistically validated.

Second, although the tool was refined through an iterative consensus process among multidisciplinary experts, it remains a decision-support framework and should not be used as a substitute for individualized clinical judgment. The subjective nature of some indicators, particularly those related to the owner’s emotional perception, may still introduce a degree of variability.

Future research should focus on the empirical validation of this checklist through large-scale clinical trials. Such studies will be essential to refine the scoring system, assess its reliability across different veterinary settings, and confirm its effectiveness in reducing caregiver burden and moral distress in both veterinarians and pet owners.

## 6. Conclusions

The management of end-of-life scenarios in dogs with CKD represents one of the most significant ethical challenges in modern veterinary practice. The holistic decision-making tool presented in this study provides a robust response to the need for methodological clarity in sensitive end-of-life moments. However, this tool should be regarded as a conceptual decision-support framework rather than a clinically validated psychometric instrument. By integrating IRIS clinical staging with an expert-reviewed three-tiered QoL scoring system, the tool shifts the focus toward a comprehensive welfare-based approach.

The classification into Tiers A, B, and C serves as a vital bridge between numerical data and ethical action. While the total score provides a quantitative baseline, the clinical focus shifts according to the specific needs of the patient and their responsiveness to treatment. It empowers veterinarians to identify the precise threshold of therapeutic futility, ensuring that euthanasia is recommended not as a default outcome of disease progression, but as a deliberate act of non-maleficence when an animal’s dignity can no longer be preserved. Furthermore, the explicit inclusion of “One Welfare” factors, such as caregiver burnout and owner resources, acknowledges the interconnectedness of human and animal well-being, providing a more sustainable and compassionate framework for shared decision-making.

Ultimately, this Decision Tree does not replace professional judgment; rather, it informs it, providing a standardized language for clinicians and a transparent roadmap for owners. Future research should focus on the large-scale clinical application of this tool to empirically refine the scoring thresholds and further evaluate its impact on reducing decisional conflict for owners. In conclusion, the adoption of such structured protocols is essential to ensure that the final stages of a dog’s life are managed with the highest standards of veterinary ethics and clinical excellence.

## Figures and Tables

**Table 1:** Specific checklist created to analyse and evaluate QoL in dogs with CKD.

Category	Guidance Questions	Score (0–2)
Pain and discomfort	Does the dog show signs of pain (e.g., vocalizations, stiff posture, anxiety)?	2 = No obvious signs of pain, normal posture, calm. 1 = Episodes of pain manageable with analgesics or mild signs. 0 = Obvious, continuous pain, uncontrollable with therapy.
2. Hunger and nutrition	Does the dog eat voluntarily? Does it maintain its body weight?	2 = Eats spontaneously, stable weight. 1 = Intermittent anorexia, need for stimulants or assisted diet. 0 = Constant refusal of food, weight loss, cachexia.
3. Hydration	Is your dog hydrated? Does it drink fluids on its own or does it need constant fluid therapy?	2 = Drinks on its own, good hydration. 1 = Occasionally requires fluid therapy or shows mild signs of dehydration. 0 = Severe dehydration, requires frequent hydration (SC or IV).
4. Hygiene and incontinence	Can you urinate independently? Do you have severe incontinence or frequently soil yourself?	2 = Independent hygiene, no leakage 1 = Occasional incontinence or soiling 0 = Severe incontinence, constantly soiled
5. Happinnes/Environmental interest	Does the dog interact with the environment, people, other animals?	2 = The dog is responsive, seeks interaction, shows curiosity towards its surroundings, responds to positive stimuli (owner’s voice, walk, favourite food), and has moments of play or peaceful rest. 1 = The dog has reduced interest, is less responsive than usual but occasionally interacts, alternates between moments of apathy and phases of attention and contact. It is not completely uninterested. 0 = The dog is completely apathetic, does not react to familiar stimuli, isolates itself, refuses contact, shows no positive emotions; may show signs of behavioural depression or uremic dementia.
6. Mobility	Can the dog stand up, walk, and move around without excessive pain?	2 = Walks, stands up, moves normally 1 = Difficulty or pain, but still mobile 0 = Immobility or unbearable pain when moving
7. Comorbidity and Multimorbidity Management	Does the dog have other chronic conditions (e.g., heart disease, osteoarthritis, diabetes)? Do these conditions complicate CKD management or limit treatment options?	2 = Not present comorbidity 1= Present comorbidity but manageable; it does not influence particularly patient’s health and does not directly conflict with renal therapy 0 = Severe or multiple comorbidities; treatments conflict (e.g., needing NSAIDs for pain which are contraindicated for kidneys) or drastically reduce prognosis.
8. Age-related prognostic Indicators	What is the patient’s age relative to breed-specific life expectancy? Does the dog show signs of “geriatric frailty” independent of renal disease? How old is the patient?	2 = Age < 7 years Young or Adult; well below the average life expectancy for the breed and size. 1 = Age from 7 to 15 years Senior; within or approaching expected lifespan; showing normal age-related changes but remains resilient. 0 = Age > 15 years Advanced Geriatric; beyond average life expectancy; high biological frailty or presence of canine cognitive dysfunction
9. Positive vs. Negative days	In the last week, did you have more “good” days or “bad” days?	2 = >70% of days are “good” (active, interactive, eating). 1 = ~50% of days are good. 0 = <50% of days are good or negative days prevail.
10. Response to treatments	Do palliative treatments (fluids, drugs, diet) produce improvements?	2 = Good response to treatment (fluids, diet, medication) 1 = Partial or inconsistent response 0 = No response or worsening
11. Clinical prognosis (IRIS stage)	Is there stage IV CRF with signs of refractory uraemia or neurological symptoms?	2 = IRIS I–II, good prognosis 1 = IRIS III, uncertain response 0 = IRIS IV advanced, terminal signs
12. Home-Care Feasibility and Suitability Index	Can the prescribed treatment plan (fluids, medications, specialized diet) be administered at home effectively without compromising the dog’s dignity or the owner’s ability to provide a safe environment, or is hospitalization required?	2 = The prescribed therapy can be comfortably administered by the owner at home. The home environment is conducive to recovery and hygiene, and the owner is fully confident in daily tasks. Home care is preferred over hospitalization to maintain the dog’s comfort. 1 = The dog shows mild resistance to handling, requiring significant time, physical effort, or occasional professional assistance to maintain stability or the owner requires significant external support/training to avoid hospitalization 0 = The patient’s clinical condition requires interventions that are only practicable within a hospital setting. Attempting home care compromises the patient’s welfare or exceeds the owner’s capabilities.
13. Emotional wellness of the owner	Is the owner able to manage the care without emotional compromise?	2 = Calm, supported, involved 1 = Increasing anxiety or fatigue 0 = Burnout, emotional inability to cope

**Table 2:** Summary of Clinical Objectives and Interventions by Tier.

Tier	Score	Clinical Status	Primary Medical Goal	Key Interventions
Tier A	20–26	Stable welfare; manageable clinical signs.	Active Palliation: Optimization of QoL.	Pharmacological control of uremic nausea/vomiting, tailored hydration protocols, renal diet.
Tier B	14–19	Ambiguous condition; fading biological resilience.	Intensive Support: Re-evaluation of therapeutic efficacy.	Frequent clinical monitoring, ethical reappraisal, in-depth owner counseling.
Tier C	0–13	Refractory suffering: therapeutic futility reached.	End-of-Life Compassion: Prevention of further distress.	Euthanasia to ensure a dignified death and avoid metabolic agony.

## Data Availability

The original contributions presented in this study are included in the article. Further inquiries can be directed to the corresponding author.
